# GDF11 restores skeletal muscle satellite cell function via MYC to ameliorate sarcopenia

**DOI:** 10.1016/j.gendis.2026.102095

**Published:** 2026-02-14

**Authors:** Feng Zhang

**Affiliations:** a Department of Geriatrics, Huashan Hospital Affiliated to Fudan University, Shanghai 200040, China; b National Clinical Research Center for Aging and Medicine, Huashan Hospital, Fudan University, Shanghai 200040, China

Sarcopenia is a prevalent age-related condition that significantly compromises the health and quality of life of elderly individuals. One of its hallmark features is the senescence and functional decline of skeletal muscle satellite cells (MuSCs), which are critical for muscle regeneration. Prior research has suggested that growth differentiation factor 11 (GDF11) may exert rejuvenating effects across various tissues, including skeletal muscle. Our previous bioinformatics studies indicated that GDF11 could suppress apoptosis, enhance proliferation and differentiation, and improve MuSC functionality. Notably, MYC was identified as a key transcription factor downstream of GDF11 signaling. In the present study, we investigated the molecular mechanisms through which GDF11 mediates MuSC rejuvenation in sarcopenia. We conducted *in vitro* experiments using GDF11, MYC overexpression, and MYC inhibition to assess their impact on MuSC biology. The results demonstrated that GDF11 notably enhanced MuSC function by facilitating cell proliferation and myogenic differentiation, reducing apoptotic activity, mitigating DNA damage, preserving telomere integrity, and improving mitochondrial performance (Fig. 1A). These effects were largely dependent on the presence of MYC. Collectively, our findings suggest that GDF11, through modulation of MYC, holds promise as a therapeutic strategy for restoring MuSC function and potentially alleviating sarcopenia.

**Figure 1:**
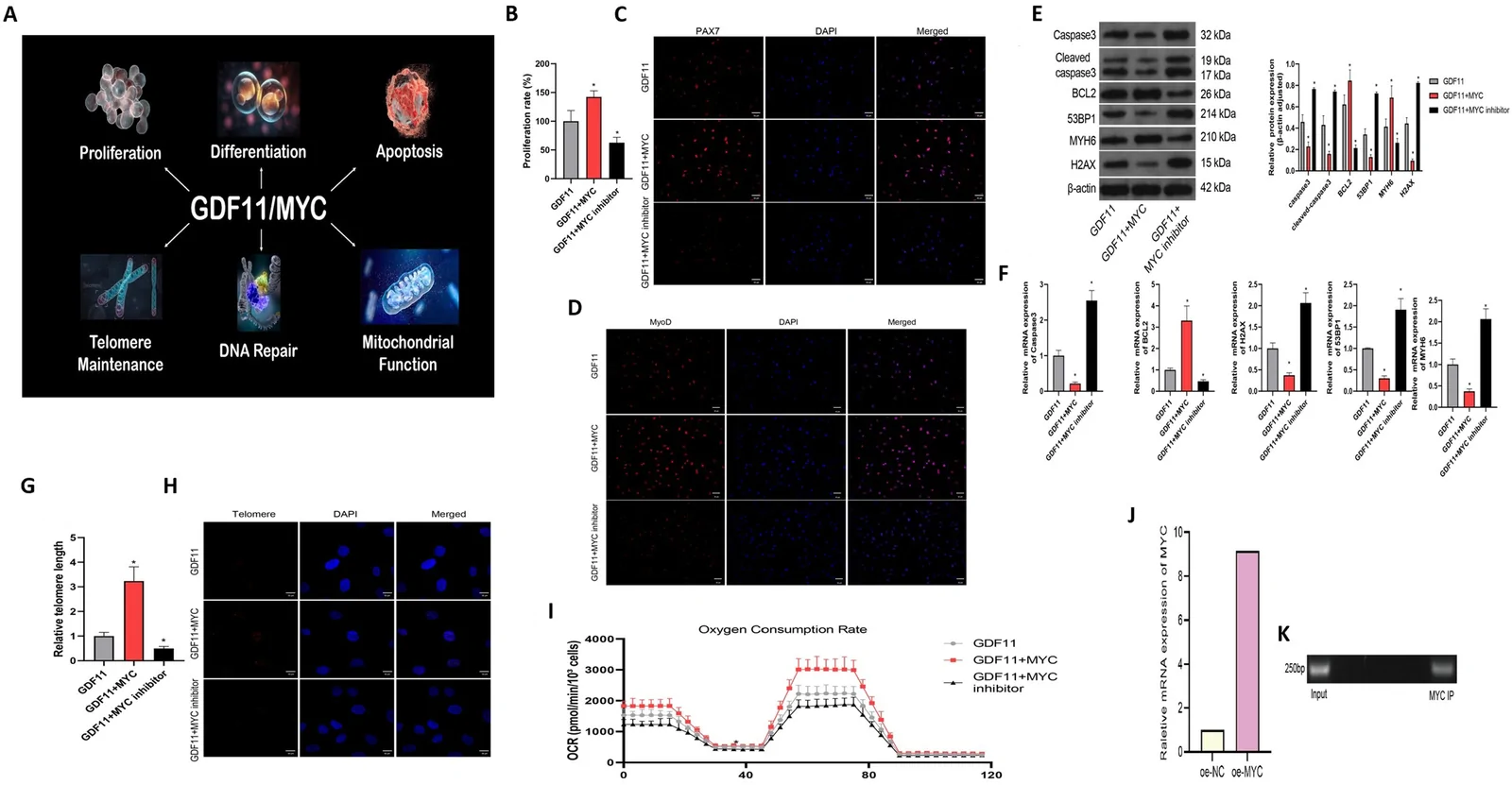
The effect of GDF11 and MYC on MuSCs (promotes proliferation and differentiation, suppresses apoptosis and DNA damage, enhances telomere length and mitochondrial oxygen consumption), and the interaction between GDF11 and MYC on MuSCs. **(A)** Graphic Abstract of GDF11 and MYC on MuSCs in Sarcopenia. **(B)** Proliferation of MuSCs treated with GDF11, GDF11+MYC, or GDF11+MYC inhibitor 10058-F4, as assessed by the CCK-8 assay (*n* = 3). Data are presented as the mean ± standard error of the mean, ∗*P* < 0.05. **(C)** Representative immunofluorescence images showing Pax7 (red) expression in MuSCs across the GDF11 group, the GDF11+MYC group, and the GDF11+MYC inhibitor group. Nuclei were counterstained with 4′,6-Diamidino-2-phenylindole (DAPI) (blue). Scale bar = 50 μm (*n* = 3). **(D)** Representative immunofluorescence images showing MyoD (red) expression in the GDF11 group, the GDF11+MYC group, and the GDF11+MYC inhibitor group. Nuclei were counterstained with 4′,6-Diamidino-2-phenylindole (DAPI) (blue). Scale bar = 50 μm (*n* = 3). **(E)** Western blotting analysis of protein expression levels associated with apoptosis (Caspase-3 and Bcl2), DNA damage (γ-H2AX and 53BP1), and muscle function (MYH6) in MuSCs following different treatments (*n* = 3). Data are presented as the mean ± standard error of the mean, ∗*P* < 0.05. **(F)** RT-qPCR analysis of the mRNA levels for *caspase-3*, *Bcl-2*, *γ-H2AX*, *53BP1,* and *MyHC* (*n* = 3). Data are presented as the mean ± standard error of the mean, ∗*P* < 0.05. **(G)** RT-qPCR analysis of relative telomere length and telomerase activity in MuSCs treated with GDF11, GDF11+MYC, or GDF11+MYC inhibitor (*n* = 3). Data are presented as the mean ± standard error of the mean, ∗*P* < 0.05. **(H)** Representative images of telomere fluorescence in situ hybridization (FISH), showing telomeres labeled with a Cy3-labeled probe (red) and nuclei counterstained with DAPI (blue). Red, telomere probe; blue, DAPI-stained nuclei. Scale bars = 20 μm (*n* = 3). **(I)** The mitochondrial oxygen consumption rate (OCR) was measured in MuSCs under different treatment conditions (*n* = 3). Data are presented as the mean ± standard error of the mean. **(J)** RT-qPCR analysis of MYC mRNA expression levels in MuSCs under different treatment conditions (*n* = 3). **(K)** Chromatin immunoprecipitation (ChIP) assay showing the binding of GDF11 to the promoter region of the *MYC* gene (*n* = 3). All experiments were independently performed in triplicate.

Sarcopenia is highly prevalent, affecting up to 24% of individuals between the ages of 65 and 70, with muscle mass declining by as much as 15% during the seventh and eighth decades of life. Sarcopenia is closely associated with several adverse health outcomes, including frailty, and increased risks of mortality, and hospitalization.^1^ GDF11 has emerged as a promising candidate for the treatment of age-related conditions, including sarcopenia.^2^ In aged skeletal muscle, the number and functional capacity of MuSCs are significantly reduced, resulting in impaired regenerative potential. MYC is a key transcription factor (TF) that plays a critical role in muscle development.^3^ Our earlier bioinformatics analyses revealed that GDF11 interacted with MYC within transcriptional regulatory networks.^4^ In this study, we probed into the effects of GDF11 and MYC on MuSCs. These results suggest that GDF11 activates a complex signaling cascade in skeletal muscle, with MYC serving as a key downstream effector to enhance MuSC functionality and mitochondrial quality control.

To analyze the potential of GDF11 and MYC in MuSC proliferation, we first performed a CCK-8 assay. As depicted in Figure 1B, co-treatment with GDF11 and MYC significantly enhanced cell viability compared to GDF11 alone, while the addition of a MYC inhibitor (10058-F4) markedly reduced cell viability relative to the GDF11+MYC group. These results suggest that MYC facilitates the pro-proliferative effect of GDF11 in MuSCs. Consistently, immunostaining for Pax7 revealed an increased number of Pax7^+^ MuSCs in the GDF11+MYC group compared to the GDF11 group, whereas Pax7^+^ cell numbers were reduced in the GDF11+MYC inhibitor group (Fig. 1C). Immunofluorescence analysis demonstrated that MyoD expression was elevated in the GDF11+MYC group compared to the GDF11 group, while a marked reduction was observed in the GDF11+MYC inhibitor group. These findings uncover that the combined action of GDF11 and MYC synergistically enhances the myogenic differentiation of MuSCs (Fig. 1D). We next focused on the effects of GDF11 and MYC on MuSC apoptosis. Compared to the GDF11 group, the GDF11+MYC group exhibited reduced expression of DNA damage and apoptosis markers, including 53BP1, cleaved caspase-3, and γ-H2AX, while the levels of BCL2 (anti-apoptotic) and MYH6 (muscle structural protein) were elevated. In contrast, combined administration of GDF11 and a MYC inhibitor markedly elevated the abundance of 53BP1, cleaved caspase-3 and γ-H2AX while concomitantly reducing BCL2 and MYH6 levels (Fig. 1E and F). These data indicate that GDF11 and MYC inhibit apoptosis and DNA-damage responses, but preserves muscle-specific protein expression in MuSCs. As illustrated in Figure 1G and H, RT-qPCR analysis revealed that telomere length and telomerase activity were increased in the GDF11+MYC group compared to the GDF11 group. In contrast, telomere length was significantly reduced in the GDF11+MYC inhibitor group, suggesting that MYC enhances the telomere-maintaining effects of GDF11. To further investigate the role of MYC in regulating mitochondrial function in MuSCs, we assessed the OCR as a measure of mitochondrial activity. As illustrated in Figure 1I, treatment with GDF11+MYC notably increased the cellular OCR compared to that of the GDF11 group, indicating enhanced mitochondrial respiration. In contrast, the GDF11+MYC inhibitor group showed a marked reduction in OCR relative to the GDF11 group, suggesting that MYC is required for the GDF11-mediated enhancement of mitochondrial function. ChIP analysis in MuSCs demonstrated that GDF11 binds with high affinity to the promoter region of the *MYC* gene (Fig. 1J and K). To validate this interaction, MuSCs were transfected with a p3xFLAG-CMV-10-Homo MYC vector to express exogenous FLAG-tagged MYC (p3x); cells transfected with the empty p3xFLAG-CMV-10 vector served as controls. Then, ChIP-seq was performed using an anti-MYC antibody. After fixation and sonication, chromatin was immunoprecipitated with the p3x antibody. A 250-bp promoter region enriched in the ChIP-seq dataset was subsequently validated by site-specific PCR amplification. All experiments were independently performed in triplicate.

Building on our previous demonstration that MYC operates as a principal GDF11 transcription factor,^5^ we now provide a comprehensive analysis of MYC in the regulation of MuSC fate. In our study, we demonstrated that MYC significantly enhanced MuSC proliferation and up-regulated the expression of muscle function-related proteins, including MYH6 and MyHC, further supporting its role in promoting both muscle regeneration and functional maturation (Fig. 1B, C, E, F). This involves the up-regulation of key myogenic regulatory factors, such as Myf5 and MyoD, which are essential for the commitment of satellite cells to the myoblast lineage. In our study, we found that MYC enhanced MuSC differentiation, as indicated by a significant increase in MyoD^+^ MuSCs following MYC overexpression (Fig. 1D). In MuSCs, mechanistically, MYC interacts with various transcriptional and signaling pathways to modulate apoptotic responses. In this study, MYC overexpression significantly reduced apoptosis in MuSCs, suggesting a protective role in maintaining cellular viability during muscle regeneration (Fig. 1E and F). Several studies have demonstrated that MYC plays an important role in promoting DNA damage repair. In our study, GDF11 in combination with MYC appeared to mitigate DNA damage in MuSCs, as indicated by decreased expression of DNA damage markers such as γ-H2AX and 53BP1 (Fig. 1E and F). MYC is a well-established transcriptional activator of telomerase reverse transcriptase (TERT), the catalytic subunit of telomerase. In our study, we observed that MYC significantly enhanced telomerase activity and extended telomere length in MuSCs (Fig. 1G and H). MYC plays a key role in regulating mitochondrial structure and bioenergetic function. MYC depletion leads to reduced expression of mitochondrial-encoded genes. In our study, MYC treatment significantly increased the OCR in MuSCs (Fig. 1I). MYC is a key TF that regulates fundamental cellular processes, including proliferation, differentiation, cell cycle progression, metabolism, and apoptosis. MYC functions as a global transcriptional amplifier with broad biological significance. Our previous research identified MYC as a critical downstream effector of GDF11 signaling in MuSCs.^4^ In the current study, ChIP assays confirmed that GDF11 binds to the promoter region of the MYC gene (Fig. 1J and K). We demonstrate that GDF11, besides canonical SMAD signaling, could directly regulate MYC by binding to the promoter, which might interact with smad nuclear interacting protein 1 (SNIP1). This interaction further enhances the transcriptional activation of MYC.^5^ These results highlight MYC as a central mediator of GDF11 signaling and suggest that targeting the GDF11–MYC axis could be a promising therapeutic strategy for sarcopenia. Collectively, our findings suggest that GDF11, through modulation of MYC, holds promise as a therapeutic strategy for restoring MuSC function and potentially alleviating sarcopenia.

## CRediT authorship contribution statement

**Feng Zhang:** Writing – original draft, Software, Resources, Funding acquisition, Formal analysis, Data curation.

## Funding

This work was supported by the Program of Shanghai Municipal Health Commission (China) (No. 202240040).

## Conflict of interests

The authors declare no competing interests.

## References

[1] Alorfi N.M., Alshehri F.S., Ashour A.M. (2025). Therapeutics for sarcopenia and functional disabilities in older adults: a review of phase 4 clinical trials. Drug Des Dev Ther.

[2] Sinha M., Jang Y.C., Oh J. (2014). Restoring systemic GDF11 levels reverses age-related dysfunction in mouse skeletal muscle. Science.

[3] Huo F., Liu Q., Liu H. (2022). Contribution of muscle satellite cells to sarcopenia. Front Physiol.

[4] Zhang F., Yang X., Bao Z. (2022). Bioinformatics network analyses of growth differentiation factor 11. Open Life Sci.

[5] Chen Y., Guo W., Guo X., Qiao W., Yin Z. (2024). The clinical utilization of SNIP1 and its pathophysiological mechanisms in disease. Heliyon.

