# Supplementary material for: GDF11 restores skeletal muscle satellite cell function via MYC to ameliorate sarcopenia
Source: Genes Dis. 2026 Feb 14;14(3):102095. doi: 10.1016/j.gendis.2026.102095 (PMC13718635; doi:10.1016/j.gendis.2026.102095)
Supplement: Supplementary file 1 [file mmc1.doc]

**Supplementary Materials**

**Methods**

**Cell culture and treatment**

Frozen human MuSCs (keycell, Hubei, CN) were thawed rapidly in a 37°C water bath and gently agitated to facilitate resuspension. Following centrifugation at 1200 rpm for 5 min, the supernatant was discarded, and the remaining cell pellets were resuspended in a complete skeletal muscle cell culture medium (QS-H822A, keycell, Hubei, CN). MuSCs were maintained in a humidified incubator at 37°C with 5% CO_2_.

The MuSCs were randomized into the following experimental groups for treatment: GDF11 group (treated with 100 ng/mL GDF11, GP20312, GLPBIO, CA, USA), GDF11+MYC group (treated with 100 ng/mL GDF11 and 100 ng/mL MYC, M2435-1MG, Sigma, MA, USA), and GDF11+MYC inhibitor group (treated with 100 ng/mL GDF11 and 100 ng/mL MYC inhibitor 10058-F4, F794822, Macklin, Shanghai, China). After complete cell attachment, treatments were administered according to group assignments for 6 days prior to subsequent experiments.

**Cell proliferation assay**

Cell proliferation was examined utilizing the Cell Counting Kit-8 (CCK-8, QS-S321, KeyCell, Hubei, China). MuSCs were seeded into 96-well plates at a density of 3 × 10^3^ cells per well and treated with GDF11, MYC, or 10058-F4 as described. After treatment, 10 μL of CCK-8 reagent was added to each well, followed by incubation at 37 °C in 5% CO_2_ for 2 h. Absorbance was measured at 450 nm using a microplate reader (Thermo Fisher Scientific, USA).

**Immunofluorescence staining**

Cells were fixed in 4% paraformaldehyde and permeabilized with PBS containing 0.25% Triton X-100 for 10 min. After blocking with 3% normal goat serum (BOSTER, Hubei, China) in PBS for 30 minutes at ambient temperature, cells were incubated overnight at 4 °C with primary antibodies diluted in PBS containing 2.5% BSA. The primary antibodies used were rabbit anti-PAX7 (1:100; Abcam, Shanghai, China) and rabbit anti-MyoD1 (1:100; Affinity, Changzhou, China). After washing, cells were incubated with a CY3-conjugated goat anti-rabbit secondary antibody (BOSTER, Hubei, China). Fluorescence imaging was performed employing a fluorescence microscope (Olympus, Japan) at 200× magnification.

**Quantitative fluorescent In Situ hybridization (Q-FISH)**

Telomere-specific fluorescent oligonucleotide probes (5′-CCCTAACCCTAACCCTAA-3′, labeled with Cy3) were synthesized by Sangon Biotech (Shanghai, China) to detect telomeric regions. MuSCs cultured for 3 days were fixed in 4% DEPC-treated paraformaldehyde for 20 min at ambient temperature. After fixation, cells were rinsed thrice with PBS (5 min per wash) and pre-hybridized for 1 h in wash buffer (2× SSC, 10% formamide) at ambient temperature. Hybridization was performed overnight (16 h) at 37 °C in the dark using 8 ng/μL of the probe in 50 μL hybridization buffer (2× SSC, 10% formamide, 10% dextran sulfate). Following hybridization, cells were rinsed with 2× SSC (Servicebio, Hubei, China) and washed with PBS for 5 min. Nuclei were stained with 0.05 μg/mL DAPI (Beyotime, Shanghai, China) for 8 min at 37 °C. Images were captured using a Nikon Eclipse TiSR fluorescence microscope equipped with a Nikon DS-U3 digital camera. Image analysis was performed employing CaseViewer software.

**Western blot analysis**

Total protein extraction was completed utilizing RIPA lysis buffer containing 1% PMSF (Servicebio, Hubei, China), followed by protein concentration determination with a BCA Assay Kit (Genebase, Guangdong, China). Approximately 40 μg of protein per sample was loaded onto a 10% SDS-PAGE gel for separation, then transferred onto PVDF membranes (Millipore, MA, USA). Membranes were blocked in blocking buffer (Servicebio) for 2 h at ambient temperature and incubated overnight with primary antibodies: H2AX (10856-1-AP; 1:2000; proteintech, Hubei, China), 53BP1 (AF5452; 1:1000; affbiotech, Jiangsu, China), BCL2 (26593-1-AP; 1:2000; proteintech, Hubei, China), Caspase3 (66470-2-Ig; 1:2000; proteintech, Hubei, China), MyH6 (22281-1-AP; 1:25000; proteintech, Hubei, China), goat anti-mouse IgG (SA00001-1; 1:10000; proteintech, Hubei, China) and goat anti-rabbit IgG (A0208; 1:10000; Beyotime, Shanghai, China). After washing five times with TBST, membranes were incubated with HRP-conjugated secondary antibodies for 2 h at ambient temperature. Following additional TBST washes, signals were visualized using ECL reagents. Band intensity was analyzed using Image-Pro Plus software. β-actin (1:20000) served as the internal loading control.

**Quantitative real-time polymerase chain reaction (qPCR)**

Total RNA was isolated utilizing TRIzol reagent (Ambion, TX, USA). First-strand cDNA synthesis was implemented utilizing the HiScript II Q Select RT SuperMix for qPCR (Vazyme, Jiangsu, China). qPCR was conducted on a ViiA 7 Real-Time PCR System (Thermo Fisher Scientific, Applied Biosystems, MA, USA). Primer sequences are listed in **Supplementary Table S1.**

**Chromatin immunoprecipitation (ChIP)**

ChIP assays were completed utilizing a commercial ChIP Assay Kit (Epigentek, NY, USA). Briefly, cells were cross-linked with 1% formaldehyde to stabilize DNA-protein interactions, and cross-linking was quenched by the addition of 125 mM glycine. Nuclei were isolated by incubation in hypotonic buffer containing protease inhibitors on ice for 30 min, followed by Dounce homogenization. Chromatin was fragmented by sonication and resuspended in a ChIP dilution buffer. Each immunoprecipitation reaction was performed with 10 μg of anti-MYC antibody (Proteintech, Hubei, China) or IgG as a negative control. Following immunoprecipitation, bound DNA was eluted and purified. Enrichment of target regions was quantified by qPCR. The primers used for ChIP-qPCR targeting the GDF11 promoter were: forward: 5′- ACAGGGTTTCACCGTGTTAGC-3′ and reverse: 5′- AGCCCTGGAGCAATAGTCAA-3′.

**Measurement of cellular oxygen consumption**

Total adenosine triphosphate (ATP) production and the relative contributions of mitochondrial oxidative phosphorylation and glycolysis were assessed using the ATP Rate Assay Kit (Agilent Technologies, CA, USA). MuSCs were plated in a 96-well Agilent Seahorse plate (Agilent Technologies, CA, USA) and allowed to adhere overnight. Prior to analysis, cells were incubated at 37 °C without CO_2_ for 1 h. Oxygen consumption rate (OCR) and extracellular acidification rate (ECAR) were measured using the Seahorse XFe96 Extracellular Flux Analyzer (Agilent, CA, USA). Data acquisition and analysis were done utilizing the XFe Wave software platform.

**Statistical analysis**

Statistical analysis was implemented utilizing GraphPad Prism 7 software. Data are detailed as mean ± standard deviation (SD). Comparisons among multiple groups were performed utilizing one-way analysis of variance (ANOVA), followed by Tukey’s multiple comparisons test for post hoc analysis. A *p*-value less than 0.05 was deemed statistical significance.

**Supplementary Table S1**

Primer sequences information

| Name | Primer | Sequence | Size |
| --- | --- | --- | --- |
| Homo b-actin | Forward | CCCTGGAGAAGAGCTACGAG | 180bp |
|  | Reverse | CGTACAGGTCTTTGCGGATG |  |
| Homo H2AX | Forward | TACCTCACCGCTGAGATCCT | 168bp |
|  | Reverse | CTGGATGTTGGGCAGGAC |  |
| Homo 53BP1 | Forward | CAGTCCCCAGAAGACCATGT | 235bp |
|  | Reverse | CTGGGAAGCAGGAGAAACAG |  |
| Homo BCL2 | Forward | GCCTTCTTTGAGTTCGGTGG | 192bp |
|  | Reverse | GAAATCAAACAGAGGCCGCA |  |
| Homo Caspase3 | Forward | ACTGGACTGTGGCATTGAGA | 162bp |
|  | Reverse | GCACAAAGCGACTGGATGAA |  |
| Homo MyHC | Forward | TTCATTGACTTTGGCATGGA | 170bp |
|  | Reverse | GGCTTCTGGAAATTGTTGGA |  |
| Telomere | Forward | GGTTTTTGAGGGTGAGGGTGAGGGTGAGGGTGAGGG | 252bp |
|  | Reverse | TCCCGACTATCCCTATCCCTATCCCTATCCCTATCCCTA |  |
